# Recovery after Delayed Surgery in a Case of Spinal Subdural Hematoma

**DOI:** 10.1155/2013/310854

**Published:** 2013-01-13

**Authors:** Pier Paolo Panciani, Claudio Cornali, Alessandro Agnoletti, Giacomo Esposito, Gabriele Ronchetti, Marco Fontanella

**Affiliations:** ^1^Department of Neuroscience, Division of Neurosurgery, University of Brescia, Piazza Spedali Civili, 25123 Brescia, Italy; ^2^Department of Neuroscience, Division of Neurosurgery, University of Torino, 10126 Torino, Italy

## Abstract

Spinal chronic subdural hematoma (SCSH) is a rare pathology usually associated with trauma or hematological alterations or is due to iatrogenic causes; rarely SCSH can be spontaneous. We report a case of a 79-year-old female who underwent a surgical evacuation of a spontaneous SCSH one year after diagnosis. She presented with a severe paraparesis and showed a considerable improvement in sensory-motor performances after surgery. The treatment of spontaneous SCSH is not well defined and universally accepted. Early surgery is mandatory in cases presenting with severe deficits. To the best of our knowledge, this is the first case showing a good outcome in a case of SCSH following a delayed surgery. In our opinion, an aggressive approach should be considered as a viable option in cases of spontaneous SCSH even after a lasting spinal cord compression.

## 1. Introduction

Spinal chronic subdural hematoma (SCSH) is an uncommon and often misdiagnosed pathology of the spinal cord. The etiology is usually related to trauma, iatrogenic causes (i.e., lumbar puncture), and hematological disorders. Rarely, the onset can be spontaneous. As a matter of fact, only 6 cases of spontaneous SCSH are reported in the literature [1, 2]. In case of spontaneous occurrence, the therapeutic attitude is not well defined and universally accepted. A conservative approach should be preferred in patients with mild neurological deficits or with surgical contraindications. On the other hand, in severe cases and when feasible, surgery allows to avoid worsening or chronic persistence of neurological deficits [2]. An early evacuation is generally suggested. In the literature only one case of delayed surgical treatment is reported. It was performed two months after diagnosis and a slight improvement of neurological deficits was observed [2]. We report a case of spontaneous SCSH. Surgery was performed one year after the occurrence of an acute spinal hematoma. Nevertheless, the patient had a significant improvement of her neurological conditions.

## 2. Case Report

In January 2009, a 79-year-old female presented to a hospital without neurosurgery service for the sudden onset of flaccid paraplegia and urinary retention. The magnetic resonance imaging (MRI) showed an acute spinal hematoma, extended from C5 to D6, causing a severe cord compression. The physicians decided a conservative treatment and the patient underwent a rehabilitative program.

**Figure 1:**
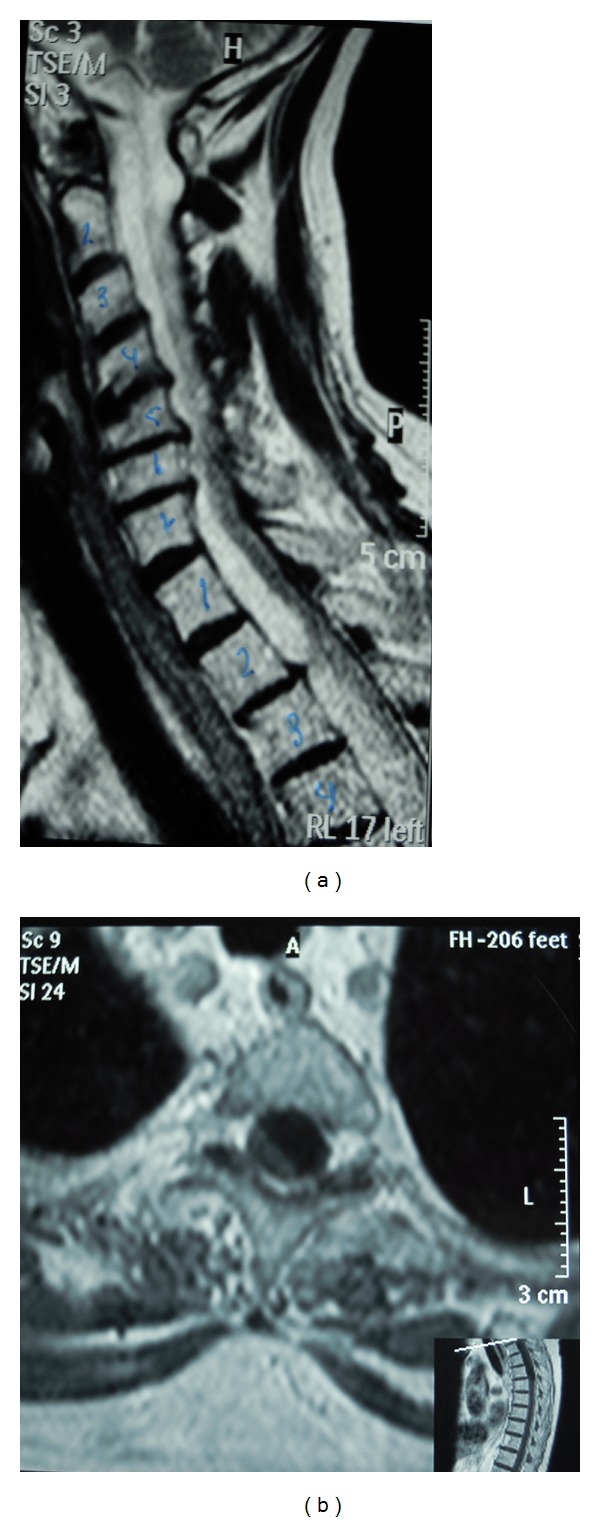
T-2 weight sagittal (a) and T-1 weight axial (b) MRI showing the cervicothoracic SCSH. A severe cord compression is observed.

She achieved a poor improvement in limb strength and sphincter functions. One year later, the patient presented to our hospital with severe paraparesis, complete anesthesia from the mammillary line, and double sphincter dysfunction. MRI showed a cervicothoracic SCSH (Figures 1(a)-1(b)). She underwent a surgical evacuation of the hematoma through D5 left hemilaminectomy. The fluid drained showed a subdural hygroma and no signs of rebleeding were found. The bacterial culture showed no signs of infection.

The sensory-motor performances improved since the first postoperative day. After one week, we observed a residual slight hyposthenia of lower limbs and a partial recovery of tactile sensitivity. The patient was discharged nine days after surgery and admitted to a rehabilitation center. After six months, the patient started to walk with a minimal assistance.

## 3. Discussion

The management of SCSH is still debated. Spontaneous spinal epidural hematoma generally requires an urgent surgical evacuation [3]. The indication of surgery for acute subdural hematoma needs to be clarified, even if the majority of cases in literature had surgical decompression [4]. On the other hand, in case of chronic subdural hematoma, especially in elderly, a conservative approach could be attempted.

In our experience, the rehabilitative program failed to ensure a long-term benefit. Therefore, we decided to perform a surgical approach that allowed to obtain, in a short time, a good recovery in sensory-motor functions. No signs of acute bleeding were found and the bacterial culture ruled out an infectious pathogenesis. 

The good outcome was achieved despite a delayed surgery. The other case of delayed surgery reported in the literature confirms the possible advantages of this management [2]. Therefore, in case of SCSH presenting with neurological deficits, we suggest an aggressive approach, when feasible, even after one year from diagnosis.
